# The association between working hours and working type with non-alcoholic fatty liver disease: results from the NHANES 1999-2014

**DOI:** 10.3389/fendo.2024.1499735

**Published:** 2025-01-14

**Authors:** Ruli Wang, Ningxi Wu, Huan Qu, Xiaowei Zheng, Haoyang Zhang, Lihong Zhu, Xiaolei Wang, Xiaodie Yao, Le Zhang

**Affiliations:** ^1^ Department of Pediatric Laboratory, Affiliated Children’s Hospital of Jiangnan University, Wuxi Children's Hospital, Wuxi, Jiangsu, China; ^2^ Department of Health Management Centre, School of Medicine, Zhongda Hospital, Southeast University, Nanjing, Jiangsu, China; ^3^ Public Health Research Center and Department of Public Health and Preventive Medicine, Wuxi School of Medicine Jiangnan University, Wuxi, Jiangsu, China

**Keywords:** working hours, working type, hepatic steatosis index, NAFLD, NHANES

## Abstract

**Background:**

Previous research has indicated that long working hours are connected to a variety of health conditions, including nonalcoholic fatty liver disease (NAFLD). However, this association which has been observed in more population is limited. Our research is designed to evaluate the association between working hours, working type, and NAFLD.

**Methods:**

The study comprised adults with complete details on working hours, working type, and NAFLD from the NHANES 1999-2014. We employed the hepatic steatosis index (HSI) to evaluate NAFLD and examined the relationship between working hours or working type and hepatic steatosis using weighted multiple-variable regression models and restricted cubic spline (RCS) analysis. In addition, further subgroup analysis was performed based on sex, age, ratio of family income to poverty (PIR), education, and diabetes.

**Results:**

Long working hours were significantly linked to an elevated risk of NAFLD (OR: 1.57, 95%CI: 1.21-2.05), even after controlling for confounding factors. RCS analysis suggested that there was no nonlinear relationship between them. When weekly working hours > 50, the likelihood of NAFLD among the population heightened to 57% and this risk increased to 99% in the female population. As for working type, increasing physical intensity of work was associated with higher NAFLD risk, but only heavy manual labor continued to show significance after adjustment (OR:1.39, 95%CI: 1.06-1.81). We observed that the relationship between heavy manual labor and NAFLD was more significant in the older and male populations.

**Conclusion:**

Our results indicate that long working hours and engaging in heavy physical labor are independent risk factors for NAFLD. As working hours increase and individuals engage in heavy physical labor for extended periods, the risk of developing NAFLD significantly rises.

## Introduction

Long working hours can have negative health consequences (1), and research has indicated that long working hours may lead to an heightened risk of hypertension (2), diabetes (3), cardiovascular disease (4, 5), obesity (6), even depression and suicidal tendencies (7). Therefore, it is crucial to plan work hours reasonably. The statutory limit on weekly work hours is less than 48 hours in most European nations (8), and about half of these nations have set a 40-hour workweek cap. Nevertheless, approximately one-third of the global labor force still works more than 48 hours per week.

It is beneficial for health to engage in exercise during free time, and it is also essential for sustaining and enhancing physical strength and work performance (9, 10). One might think that physically demanding work has an advantageous impact on health (11). However, the physical demands of work may actually be detrimental. Reports suggest that jobs with high physical demands are linked to greater levels of disability, reduced body function, and decreased in muscle power (12–14).The differences in physical activity patterns between work and leisure time might be a crucial factor in clarifying this occurrence (15). Furthermore, intense manual labor could contribute to a lack of exercise during leisure time (16).

Non-alcoholic fatty liver disease (NAFLD) is a prevalent metabolic condition with an increasing global incidence and has become a significant factor in chronic liver disease in many parts of the world (17, 18). As people’s understanding of the disease deepens, it has been discovered that NAFLD is actually a disease related to metabolic dysfunction. It was renamed metabolic dysfunction-associated fatty liver disease (MAFLD) in 2020 and subsequently renamed metabolic dysfunction-associated steatotic liver disease (MASLD) in 2023, and a new category, metabolic dysfunction and alcohol-associated liver disease (MetALD), was proposed (19–21). The natural course of NAFLD includes a wide range of pathological conditions, from simple steatosis to steatohepatitis (NASH), as well as with varying levels of fibrosis and cirrhosis (22). Many factors are associated with NAFLD, including obesity, diabetes, hypertension, and dyslipidemia (23, 24). The main cause of death in patients with NAFLD is cardiovascular disease (25). NAFLD is becoming a more significant public health challenge (17), making prevention crucially important.

Both long working hours and heavy physical labor may impact people’s health, therefore we aim to analyze the connection between working hours, working type, and NAFLD. Previous studies have shown that working long hours is strongly linked to NAFLD (26), but they did not indicate whether the association is linear or a dose-response. Additionally, the Korean data may not be representative of the situation in other regions, as it only represents a subset of the Asian population. This study aims to expand the population to include the United States to understand whether the link between long working hours and NAFLD exists in this context. Additionally, no one has explicitly studied the relationship between working type and NAFLD. We will classify occupations based on work intensity to investigate whether different types of occupations are associated with NAFLD.

## Methods

### Research design and subjects

**Figure 1:**
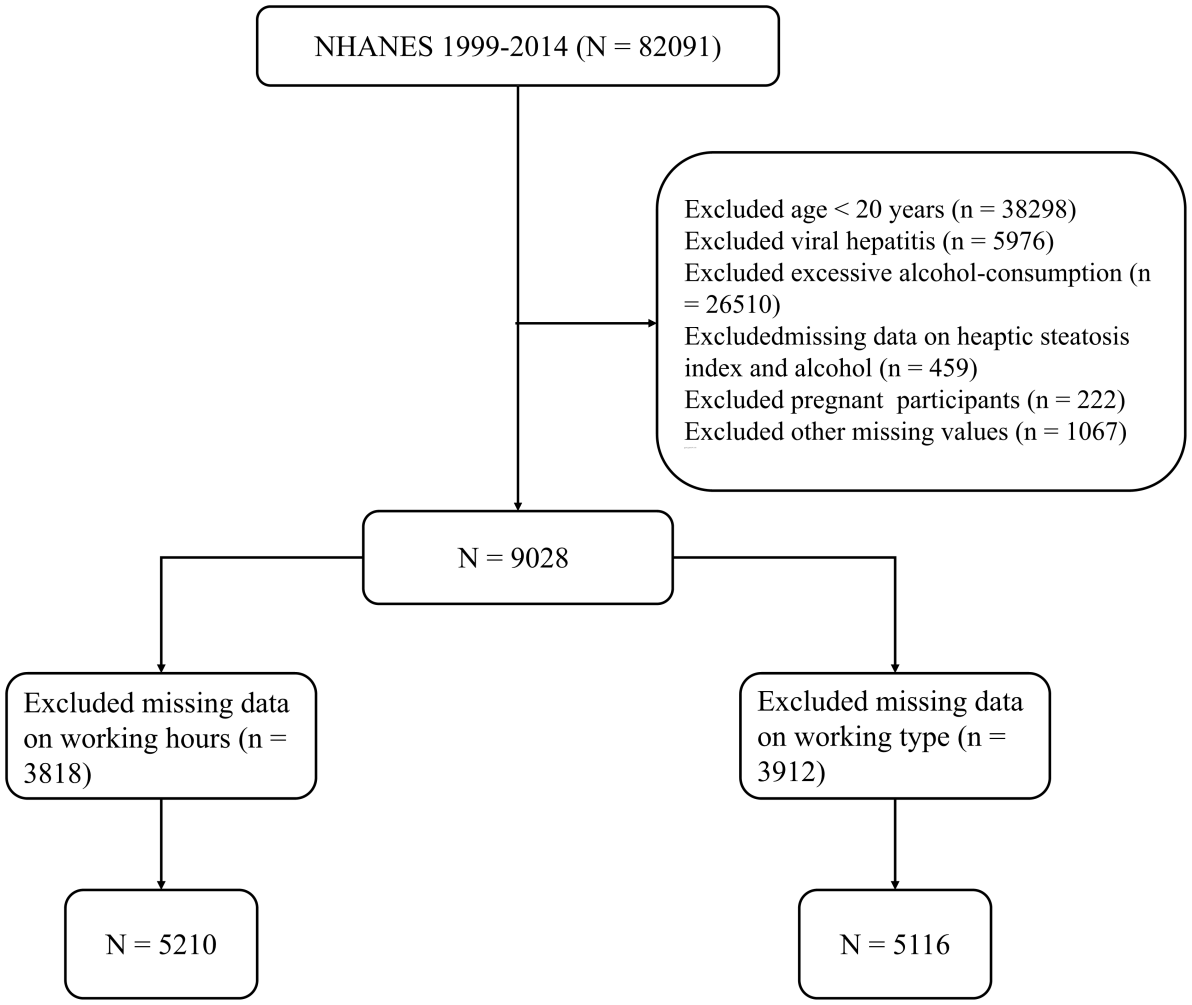
Flowchart of participants selection.

Data were obtained from the National Health and Nutrition Examination Survey (NHANES), which aims to evaluate the health and nutritional status of both children and adults in the United States (27, 28). We screened 43793 participants aged 20 years and older, which was collected from the NHANES 1999-2014. Exclusion criteria included: (1) other current hepatic disorders or factors leading to chronic liver disease, including hepatitis resulting from hepatitis B virus (HBV), hepatitis c virus (HCV), and liver damage caused by iron overload and liver tumors (n = 5976); (2) individuals who consume more than 1 alcoholic drink per day for women or 2 for men (n = 3711); (3) missing data on the hepatic steatosis index (HSI) and alcohol (n = 459), (4) pregnant participants (n = 222). (5) other missing values (n = 1067). Finally, we filtered out 5210 participants with working hours data and 5116 participants with working type. **Figure 1** shows the flow of participants. The protocol was approved by National Center for Health Statistics (NCHS) Research Ethics Review Board, and all participants provided informed consent. Detailed information can be found at https://www.cdc.gov/nchs/nhanes/.

### Survey and laboratory analyses

Basic demographic features included age, sex, race (non-Hispanic White, non-Hispanic Black, Mexican American, or other), ratio of family income to poverty (PIR), education level (less than high school, high school and some college or above), body mass index (BMI), systolic blood pressure (SBP), diastolic blood pressure (DBP), smoking status, and diabetes. Alanine aminotransferase (ALT), aspartate aminotransferase (AST), total cholesterol (TC), and triglycerides (TG) were extracted. The SBP and DBP data were obtained as the average of three measurements, while data on smoking and diabetes were collected from questionnaires. Detailed laboratory testing methods such as TC, TG, ALT, AST can be found at: https://www.cdc.gov/nchs/data/series/sr_01/sr01_056.pdf. The PIR was used to categorize family income levels into low (PIR< 1.3), middle (PIR: 1.3–3.5), and high (PIR > 3.5) groups. The definition of smoking was established as having smoked 100 or more cigarettes in one’s lifetime or being a current smoker. Diabetes was defined as being informed by a doctor of having diabetes or having a fasting blood glucose concentration of 126 mg/dL or more. We classified working hours into the following categories: less than 30 hours per week, 30-40 hours, 40-50 hours, and more than 50 hours. Working type was classified into four categories: mental labor, light physical labor, medium physical labor, and heavy physical labor (**Supplementary Table S1**). We used the HSI (29) to diagnose NAFLD in this study due to the absence of abdominal ultrasound data:

$HSI=\frac{\text{alanine aminotransferase }\left(\text{ALT}\right)}{\text{aspartate aminotransferase }\left(\text{AST}\right)\;}\;$ +body mass index (BMI) +2 (if diabetic) + 2 (if female).

Prior research has shown a strong correlation between NAFLD and the extent of hepatic steatosis; therefore, we define NAFLD as an HSI greater than 36 (30).

### Statistical analysis

In descriptive analysis, data for categorical variables were reported as frequency (percentages), while continuous variables were reported as medians (IQR) due to their skewed distribution. Kruskal-Wallis tests were conducted to compare differences in continuous variables among groups, while categorical variable differences were examined using chi-square tests. Multivariate logistic regression incorporating weights was used to evaluate the association between working hours or working type and NAFLD across different models. Model 1: No adjustment was made for confounding variables. Model 2: Consideration was given to age, sex, race, PIR, and education in the adjustments. Model 3: Based on model 2, additional factors were considered, including ALT, TC, TG, DBP, SBP, smoking status, and diabetes. We employed restricted cubic spline (RCS) analysis to examine the potential non-linear relationship between working hours and NAFLD. Subgroup analysis was conducted to explore the relationship between working hours or type and NAFLD. Stratification factors included gender (male/female), age (≤ 40/40-60/> 60years), PIR (≤ 1.3/1.3-3.5/> 3.5), education level (less than high school, high school and some college or above), and diabetes (yes/no). We conducted interaction analysis to examine the heterogeneity of the relationship among various subgroups. All of our data analyses were conducted using R version 4.3.2. A two-sided *P* value less than 0.05 was considered statistically significant.

## Results

### Baseline characteristics of participants

After excluding participants with significant alcohol intake, viral hepatitis, other liver conditions, and those who missed key parameters, we identified 5210 participants for the working hours analysis and 5116 participants for the working type analysis. Our study found that for working hours, the average age of the total population was 45.4 years, with males accounting for 62.7%. In terms of working type, the average age of participants was 57.0 years, with males accounting for 57.3%.

The working hours showed significant differences among participants based on sex, age, race, education level, PIR, and ALT. As working hours increased, the proportion of males gradually increased. The median age was 50.0 years in the ≤ 30 hours group, 44.0 years in the 30-40 hours group, 45.0 years in the 40-50 hours group, and 44.0 years in the > 50 hours group. This indicates that younger participants may work longer hours than middle-aged and older participants. In the ≤ 30 hours group, 24.6% of participants were from low-income households, 32.2% from middle-income households, and 43.2% from high-income households. In the > 50 hours group, only 13.6% of participants were from low-income households, 27.9% from middle-income households, and 58.4% from high-income households. The proportion of smokers and individuals with diabetes was higher in the< 30 hours work duration group (**Table 1**).

**Table 1:** Baseline characteristics of participants in the NHANES 1999-2014 cycles.

Characteristics	Working hours ^a^				*P*
	≤ 30 (n = 965)	30-40 (n = 2094)	40-50 (n = 1227)	> 50 (n = 924)	
Sex, n (%)					**< 0.001**
Male	485 (50.3)	1221 (58.3)	848 (69.1)	712 (77.1)	
Female	480 (49.7)	873 (41.7)	379 (30.9)	212 (22.9)	
Age, median (IQR), years	50.0 (33.0, 64.0)	44.0 (34.0, 54.0)	45.0 (36.0, 54.0)	44.0 (34.0, 54.0)	**< 0.001**
Race, n (%)					**< 0.001**
Mexican American	107 (11.1)	315 (15.0)	165 (13.4)	117 (12.7)	
Non-Hispanic White	573 (59.4)	972 (46.4)	721 (58.8)	495 (53.6)	
Non-Hispanic Black	161 (16.7)	472 (22.5)	171 (13.9)	187 (20.2)	
Other	124 (12.8)	335 (16.0)	170 (13.9)	125 (13.5)	
Education, n (%)					**< 0.001**
Less than high school	308 (31.9)	733 (35.0)	341 (27.8)	275 (29.8)	
High school	310 (32.1)	657 (31.4)	345 (28.1)	282 (30.5)	
Some college or above	347 (36.0)	704 (33.6)	541 (44.1)	367 (39.7)	
Family income ^b^, n (%)					**< 0.001**
Low	237 (24.6)	327 (15.6)	135 (11.0)	126 (13.6)	
Medium	311 (32.2)	725 (34.6)	313 (25.5)	258 (27.9)	
High	417 (43.2)	1042 (49.8)	779 (63.5)	540 (58.4)	
BMI (kg/m^2^), median (IQR)	26.9 (23.5, 30.9)	27.5 (24.3, 31.6)	27.4 (24.6, 30.8)	27.6 (24.7, 32.1)	0.466
TC (mmol/L), median (IQR)	5.0 (4.4, 5.7)	5.0 (4.4, 5.7)	5.1 (4.5, 5.7)	5.0 (4.4, 5.7)	0.532
TG (mmol/L), median (IQR)	1.3 (0.8, 2.0)	1.3 (0.8, 1.9)	1.3 (0.9, 1.9)	1.3 (0.8, 2.1)	0.666
ALT (U/L), median (IQR)	20.0 (16.0, 27.0)	22.0 (17.0, 30.0)	23.0 (18.0, 31.0)	23.0 (18.0, 32.0)	**< 0.001**
AST (U/L), median (IQR)	23.0 (20.0, 27.0)	23.0 (20.0, 27.0)	23.0 (20.0, 28.0)	23.0 (20.0, 28.0)	0.506
SBP (mmHg), median (IQR)	120.0 (110.0, 134.0)	118.0 (110.0, 130.0)	118.0 (110.0, 128.0)	118.0 (110.0, 128.0)	0.066
DBP (mmHg), median (IQR)	70.0 (64.0, 78.0)	72.0 (66.0, 78.0)	72.0 (66.0, 80.0)	72.0 (66.0, 80.0)	0.255
Smoking, n (%)					0.061
No	579 (60.0)	1333 (63.7)	769 (62.7)	547 (59.2)	
Yes	386 (40.0)	761 (36.3)	458 (37.3)	377 (40.8)	
Diabetes, n (%)					**0.007**
No	863 (89.4)	1934 (92.4)	1145 (93.3)	854 (92.4)	
Yes	102 (10.6)	160 (7.6)	82 (6.7)	70 (7.6)	

^a^ working hours/week; ^b^ Categorized into the following 3 levels based on the family poverty income ratio: low income (≤ 1.3), medium income (1.3 to 3.5), and high income (> 3.5). BMI, body mass index; TC, total cholesterol; TG, triglyceride; ALT, alanine aminotransferase; AST, aspartate transaminase; SBP, systolic blood pressure; DBP, diastolic blood pressure; IQR, interquartile range.

Bold indicates significant results.

### Association between different working hours and NAFLD

**Figure 2:**
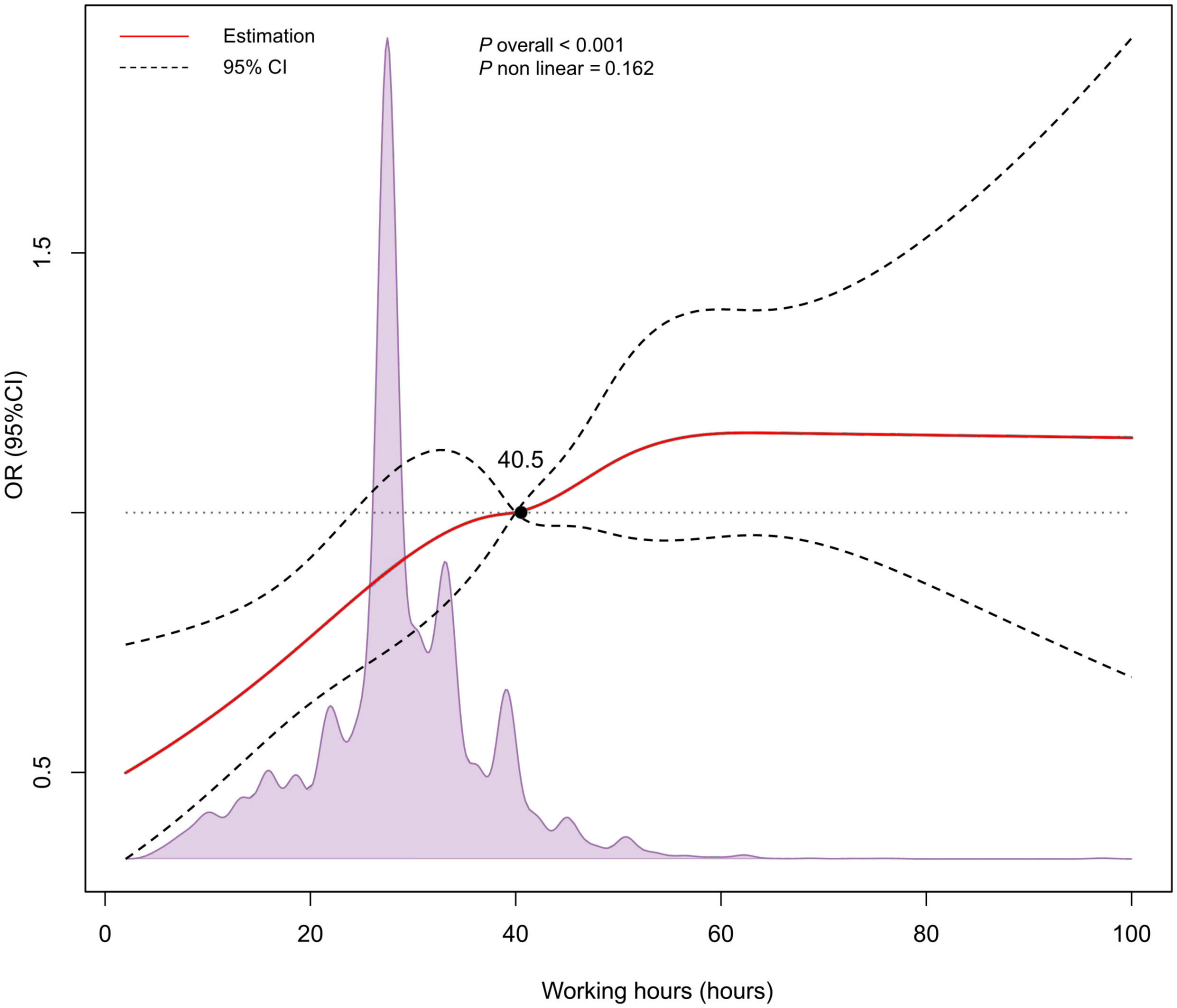
Restricted cubic spline (RCS) analysis for the relationship between working hours and NAFLD.

Compared to the reference group (≤ 30 hours), the risk of NAFLD increased with longer working hours after adjusting for confounding factors, with the significant increase in the > 50 hours group (OR: 1.57, 95%CI: 1.21-2.05, *P* = 0.006) (**Table 2**). We found that even after adjusting for age, gender, race, education level, household income, TC, TG, SBP, DBP, ALT, smoking, and diabetes, the relationship between working hours and NAFLD still persisted. In the RCS analysis, no significant nonlinear association of working hours with NAFLD was found (*P*_nonlinear_ = 0.162), but a linear correlation may exist (**Figure 2**).

**Table 2:** The association between working hours and NAFLD in participants from NHANES 1999-2014.

Characteristic	Working hours						
	≤ 30	30-40		40-50		> 50	
		OR (95%CI)	*P*	OR (95%CI)	*P*	OR (95%CI)	*P*
Model 1^a^	Ref.	**1.37 (1.14, 1.65)**	**0.001**	**1.27 (1.01, 1.60)**	**0.039**	**1.44 (1.11, 1.87)**	**0.006**
Model 2^b^	Ref.	**1.45 (1.20, 1.77)**	**< 0.001**	**1.42 (1.12, 1.81)**	**0.004**	**1.61 (1.21, 2.14)**	**0.001**
Model 3^c^	Ref.	**1.38 (1.12, 1.69)**	**0.003**	**1.36 (1.06, 1.75)**	**0.017**	**1.57 (1.21, 2.05)**	**0.001**

^a^ unadjusted; ^b^ adjusted for age, sex, race, educational level, family income; ^c^ Adjusted for Model 2, smoking, diabetes, total cholesterol, triglyceride, systolic blood pressure, diastolic blood pressure, alanine aminotransferase. NAFLD, nonalcoholic fatty liver disease.

Bold indicates significant results.

### Relationship between working type and NAFLD

The type of work was significantly associated with NAFLD before adjusting for confounding factors, and the risk of developing NAFLD increased with higher work intensity. In light physical labor, the probability of NAFLD heightened by 33% (OR: 1.33, 95%CI: 1.08-1.65, *P* = 0.008). In medium physical labor, the risk heightened by 62% (OR: 1.62, 95%CI: 1.26-2.08, *P*< 0.001). For heavy physical labor, the risk heightened by 76% (OR: 1.76, 95%CI: 1.44-2.51, *P*< 0.001). After correcting for age, sex, race, education level, and PIR, no significant connection existed between light physical labor and NAFLD (*P* = 0.299). Medium physical labor and heavy physical labor were significantly associated with NAFLD. Heavy physical labor showed a 37% increased risk (OR: 1.37, 95%CI: 1.07-1.76, *P* = 0.014). Further adjustment for TC, TG, SBP, DBP, ALT, smoking status, and diabetes showed that this significant association was only observed in heavy physical labor (OR: 1.39, 95%CI: 1.06-1.81, *P* = 0.018). Additionally, there was a tendency toward an association between medium physical labor and NAFLD (*P* = 0.087) (**Table 3**).

**Table 3:** The association between working type and NAFLD in participants from NHANES 1999-2014.

Characteristic	Working type						
	Mental labor	Light physical labor		Medium physical labor		Heavy physical labor	
		OR (95%CI)	*P*	OR (95%CI)	*P*	OR (95%CI)	*P*
Model 1^a^	Ref.	**1.33 (1.08, 1.65)**	**0.008**	**1.62 (1.26, 2.08)**	**< 0.001**	**1.76 (1.44, 2.15)**	**< 0.001**
Model 2^b^	Ref.	1.13 (0.90, 1.41)	0.299	**1.33 (1.02, 1.74)**	**0.037**	**1.37 (1.07, 1.76)**	**0.014**
Model 3^c^	Ref.	1.18 (0.92, 1.51)	0.194	1.30 (0.96, 1.75)	0.087	**1.39 (1.06, 1.81)**	**0.018**

^a^ unadjusted; ^b^ adjusted for age, sex, race, educational level, family income; ^c^ adjusted for Model 2, smoking, diabetes, total cholesterol, triglyceride, systolic blood pressure, diastolic blood pressure, alanine aminotransferase.

Bold indicates significant results.

### Relationship between working hours and NAFLD in various subgroups

In subgroup analyses stratified by sex, our findings indicated a notable positive correlation between working hours and NAFLD in females (*P<* 0.050), but no statistically association was detected in male models. There was no statistically association between working hours and NAFLD in the age-stratified analysis. In subgroup analyses stratified by family income, the risk of NAFLD increased in medium-income and high-income populations. In analysis stratified by education level, it is apparent that longer working hours are independently correlated with NAFLD in individuals with a high school education or above (*P<* 0.050), but not in those with lower educational levels. Among people without diabetes, there is a significant association between working hours and NAFLD (**Table 4**).

**Table 4:** Associations of working hours in various subgroups among participants with NAFLD in NHANES 1999-2014.

Characteristic	Working hours				*P* for interaction
	≤ 30	30-40	40-50	> 50	
Sex					0.181
Male	Ref.	1.15 (0.89, 1.47)	1.21 (0.93, 1.58)	1.26 (0.96, 1.66)	
Female		**1.61 (1.25, 2.10)**	**1.69 (1.24, 2.30)**	**1.99 (1.38, 2.89)**	
Age ^a^					0.338
Young	Ref.	1.27 (0.95, 1.72)	1.30 (0.93, 1.84)	1.27 (0.89, 1.82)	
Middle		1.21 (0.91, 1.62)	1.30 (0.95, 1.77)	**1.60 (1.15, 2.23)**	
Old		1.44 (0.99, 2.09)	1.54 (0.97, 2.46)	1.46 (0.85, 2.54)	
PIR ^b^					0.556
Low	Ref.	1.28 (0.86, 1.91)	1.16 (0.69, 1.93)	1.16 (0.69, 1.96)	
Medium		1.32 (0.97, 1.81)	**1.45 (1.00, 2.09)**	**1.85 (1.25, 2.75)**	
High		**1.45 (1.11, 1.88)**	**1.51 (1.15, 1.99)**	**1.50 (1.11, 2.04)**	
Education					0.823
Less than high school	Ref.	1.13 (0.83, 1.54)	1.10 (0.77, 1.57)	1.11 (0.75, 1.63)	
High school		**1.56 (1.14, 2.14)**	**1.77 (1.23, 2.55)**	**1.87 (1.28, 2.76)**	
Some College or above		**1.42 (1.05, 1.93)**	**1.49 (1.08, 2.05)**	**1.70 (1.20, 2.42)**	
Diabetes					0.696
No	Ref.	**1.37 (1.14, 1.65)**	**1.44 (1.17, 1.77)**	**1.50 (1.21, 1.88)**	
Yes		0.82 (0.33, 1.97)	0.73 (0.27, 1.95)	1.64 (0.54, 5.41)	

^a^ Age was classified into 3 groups: 40 years or younger, 41to 60 years and 61 years or older. ^b^ Categorized into the following 3 levels based on the family poverty income ratio: low income (≤ 1.3), medium income (1.3 to 3.5), and high income (> 3.5).

Bold indicates significant results.

### Association between working type and NAFLD in various subgroups

In subgroup analyses stratified by sex, men were at greater risk for NAFLD with medium physical labor and heavy physical labor, while no significant difference was observed in females. In subgroup analyses stratified by age, NAFLD risk increased among older individuals with higher work intensity (OR: 1.49, 95%CI: 1.11-2.00). In the family income layered analysis, we did not detect a correlation between the type of work and NAFLD. There was no significant difference in the risk of NAFLD among individuals with a high school education or below in light and moderate physical labor. Finally, in the diabetes stratified analysis, there was no meaningful connection between the type of work and NAFLD. Furthermore, the results of the interaction analysis indicated no notable interaction between working hours and the various subgroups (**Table 5**).

**Table 5:** Associations of working type in various subgroups among participants with NAFLD in NHANES 1999-2014.

Characteristic	Working type				*P* for interaction
	Mental labor	Light physical labor	Medium physical labor	Heavy physical labor	
Sex					0.593
Male	Ref.	1.27 (0.95, 1.69)	**1.73 (1.26, 2.38)**	**1.55 (1.17, 2.07)**	
Female		1.01 (0.78, 1.30)	1.07 (0.79, 1.45)	1.24 (0.87, 1.76)	
Age ^a^					0.840
Young	Ref.	0.86 (0.55, 1.34)	1.14 (0.73, 1.78)	0.97 (0.59, 1.58)	
Middle		1.14 (0.78, 1.66)	1.37 (0.90, 2.09)	1.36 (0.90, 2.05)	
Old		1.26 (0.97, 1.63)	**1.69 (1.23, 2.33)**	**1.49 (1.11, 2.00)**	
PIR ^b^					0.617
Low	Ref.	0.53 (0.31, 0.88)	0.96 (0.58, 1.60)	0.88 (0.53, 1.45)	
Medium		1.14 (0.83, 1.57)	1.42 (0.99, 2.02)	**1.52 (1.08, 2.15)**	
High		**1.33 (1.01, 1.75)**	1.39 (0.98, 1.97)	1.37 (0.98, 1.93)	
Education					0.211
Less than high school	Ref.	0.60 (0.38, 0.95)	1.01 (0.63, 1.63)	0.87 (0.55, 1.37)	
High school		1.16 (0.84, 1.62)	1.33 (0.91,1.95)	**1.56 (1.07, 2.28)**	
Some College or above		**1.33 (1.00, 1.77)**	1.34 (0.93, 1.92)	1.42 (0.95, 2.10)	
Diabetes					0.085
No	Ref.	1.12 (0.78, 1.61)	1.43 (0.96, 2.13)	1.45 (0.98, 2.15)	
Yes		1.22 (0.16, 8.05)	1.50 (0.15, 13.27)	0.26 (0.03, 1.72)	

^a^ Age was classified into 3 groups: 40 years or younger, 41 to 60 years and 61 years or older. ^b^ Categorized into the following 3 levels based on the family poverty income ratio: low income (≤ 1.3), medium income (1.3 to 3.5), and high income (> 3.5).

Bold indicates significant results.

## Discussion

In this study, we found that long working hours significantly increased the risk of NAFLD even after adjusting for confounding factors. Previous research indicated that long working hours raise the likelihood of NAFLD among Koreans (26). In our study, we expanded the population to the United States and found that working hours continued to show a significant relationship with NAFLD even after adjusting for confounding variables, which is consistent with the findings in Koreans. The risk in the US population (before correcting for confounding variables) was relatively higher (OR: 1.57, 95%CI: 1.21-2.05) compared to Korea. This may be attributed to the higher prevalence of NAFLD in North America (31.2%) compared to East Asia (29.7%) (31). Using unconstrained cubic splines based on the Korean study, we demonstrated a linear relationship between working hours and NAFLD (*P*_nonlinear_ = 0.162) (26). A Chinese study showed that prolonged and high-frequency night shift work increases the risk of NAFLD in male steel workers by 27% (32). Furthermore, the 2024 study by Robert Maidstone et al. on biobanks in the UK demonstrates that long-term night shift work elevates the risk of fatty degeneration by 8% (33). These findings are consistent with our research. Night shift work has been shown to be associated with various liver diseases. The study by Wang Feng et al. indicates that night shift work among Chinese workers is positively correlated with liver function abnormalities (34). Similarly, a study involving the Korean population found that night shift work is positively correlated with NAFLD among young female workers with poor sleep quality (35). Moreover, prolonged night shift work increases the risk of dyslipidemia and liver and kidney function abnormalities among nurses (36). Since there is no data on night shifts in the NHANES database, we used weekly working hours to investigate its association with NAFLD. These studies consistently indicate that poor work patterns can lead to the occurrence of various liver diseases.

Currently, there is no research indicating that heavy physical labor increases the risk of NAFLD. Existing studies have shown that heavy physical labor raises the likelihood of work absence (37, 38), musculoskeletal diseases (39), hypertension (40), cardiovascular disorders (41, 42), and even the risk of mortality from all causes in men (43). In addition, moderate-intensity aerobic exercise can lead to a 2-4% absolute reduction in liver fat degeneration in adults with MAFLD (44). We considered that heavy physical labor may affect the risk of NAFLD through some underlying mechanisms. To verify this hypothesis, we categorized participants into groups based on work intensity: mental labor, light manual work, moderate manual work, and heavy manual work. We observed that the risk of NAFLD increased with greater work intensity. This indirectly suggests that work-related exercise does not provide equivalent health benefits compared to free-time physical activity (45). When adjusting for age, sex, race, education level, PIR, TC, TG, SBP, DBP, ALT, smoking, and diabetes, only heavy physical labor was notably linked to NAFLD (OR: 1.39, 95%CI: 1.06-1.81). One possible explanation for this phenomenon is that while appropriate physical activity can improve mood, excessive physical activity may not only fail to enhance mood, but can also lead to mood deterioration (46, 47). This deterioration can manifest as sleep disturbances, weight and appetite loss, fatigue, irritability, emotional instability, and even depression. Sleep disturbances (48), emotional instability, and depression (49, 50) have been established as risk factors for NAFLD. Additionally, heavy manual work may contribute to a lack of leisure exercise, which is vital for promoting health and enhancing physical fitness and work capacity (9, 51). Furthermore, heavy physical labor can increase the cardiovascular burden on male construction workers, significantly raising the incidence of NAFLD (52). The specific mechanisms still need to be confirmed through extensive research.

Our subgroup analysis showed that the relationship between working hours and NAFLD was more significant in women and high-income individuals. The distribution of body fat in women changes with hormonal cycles compared to men (53), which results in a relatively higher probability of obesity in women (54). This physiological difference may contribute to an increased risk of NAFLD in women. The connection between working type and NAFLD was more pronounced in men and the elderly. This may be due to the higher proportion of male workers in heavy physical labor, as age increases, the consequences of prolonged heavy physical work become more apparent (55, 56).

Long working hours and heavy physical labor are associated with the development of various diseases. Research indicates that for every 10-hour increase in weekly working hours, the likelihood of sleep deprivation increases by approximately 50%, and the risk of difficulty falling asleep also rises significantly (57). Long working hours can lead to an increased probability of obesity (6), which may be caused by the impact of prolonged work on metabolic response mechanisms (58). Existing evidence indicates that long working hours contribute to coronary heart disease, stroke, hypertension, depression, and other chronic diseases (59). The cumulative effect of these factors greatly increases the risk of developing NAFLD. Therefore, it is crucial to properly arrange working hours and avoid intense physical labor.

However, certain limitations exist. Firstly, because this study employed a cross-sectional design, the observed connection does not inherently indicate a cause-and-effect connection. Secondly, we employed a non-direct method (the HSI assessment tool) instead of imaging studies or pathological assessments to determine NAFLD. However, the HSI has been thoroughly confirmed and can be used to predict the existence and severity of NAFLD in numerous extensive studies (29). Thirdly, since our research only consisted of the demographic in the United States, our findings ought to be corroborated in various racial groups. Ultimately, even though we tried to adjust for several potential risk variables, there could still be unaccounted confounding factors or biases beyond our control, such as participants’ specific sleep habits, daily exercise time, and dietary habits. Finally, we investigated the relationship between working hours and NAFLD; however, the associations of other steatotic liver disease categories, such as MASLD and MetALD, remain unexplored. We hope to further explore the connections between working hours, working type and other liver diseases in future research. Regardless of these limitations, this study offers important strengths, such as a large representative sample from across the nation, standardized exceptional clinical and laboratory data gathering, and thorough details on different confounding influences.

## Conclusion

The findings of this cross-sectional study suggest significant connection between long working hours and heavy physical labor with NAFLD. Furthermore, our research indicates that these factors heighten the likelihood of developing NAFLD, which may offer insights into innovative interventions and approaches for reducing the risk of NAFLD.

## Data Availability

Publicly available datasets were analyzed in this study. This data can be found here: https://www.cdc.gov/nchs/nhanes/.
