# Supplementary material for: The association between working hours and working type with non-alcoholic fatty liver disease: results from the NHANES 1999-2014
Source: Front Endocrinol (Lausanne). 2025 Jan 14;15:1499735. doi: 10.3389/fendo.2024.1499735 (PMC11772206; doi:10.3389/fendo.2024.1499735)
Supplement: Supplementary file 1 [file Table1.doc]

**Supplementary Material**

| **Table S1. The classification of working type.** | | |
| --- | --- | --- |
| **Working type** | **1999-2004** | **2005-2014** |
| Mental labor | Engineers, architects and scientists | Business, Financial Operations Occupations |
|  | Health diagnosing, assessing and treating occupations | Computer, Mathematical Occupations |
|  | Teachers | Life, Physical, Social Science Occupations |
|  |  | Legal Occupations |
|  |  | Education, Training, Library Occupations |
|  |  | Arts, Design, Entertainment, Sports, Media Occupations |
|  |  | Healthcare Practitioner, Technical Occupations |
|  |  | Healthcare Support Occupations |
| Light physical labor | Executive, administrators, and managers | Management Occupations |
|  | Management related occupations | Office, Administrative Support Occupations |
|  | Writers, artists, entertainers, and athletes |  |
|  | Other professional specialty occupations |  |
|  | Technicians and related support occupations |  |
|  | Supervisors and proprietors, sales occupations |  |
|  | Sales representatives, finance, business, & commodities ex. Retail |  |
|  | Sales workers, retail and personal services |  |
|  | Secretaries, stenographers, and typists |  |
|  | Information clerks |  |
|  | Material recording, scheduling, and distributing clerks |  |
|  | Miscellaneous administrative support occupations |  |
| Medium physical labor | Protective service occupations | Community, Social Services Occupations |
|  | Waiters and waitresses | Protective Service Occupations |
|  | Cooks | Food Preparation, Serving Occupations |
|  | Miscellaneous food preparation and service occupations | Personal Care, Service Occupations |
|  | Health service occupations | Sales & Related Occupations |
|  | Cleaning and building service occupations |  |
|  | Personal service occupations |  |
| Heavy physical labor | Farm operators, managers, and supervisors | Architecture, Engineering Occupations |
|  | Farm and nursery workers | Building & Grounds Cleaning, Maintenance Occupations |
|  | Related agricultural, forestry, and fishing occupations | Farming, Fishing, Forestry Occupations |
|  | Vehicle and mobile equipment mechanics and repairers | Construction, Extraction Occupations |
|  | Other mechanics and repairers | Installation, Maintenance, Repair Occupations |
|  | Construction trades | Production Occupations |
|  | Extractive and precision production occupations | Transportation, Material Moving Occupations |
|  | Textile, apparel, and furnishings machine operators | Armed Forces |
|  | Machine operators, assorted materials |  |
|  | Fabricators, assemblers, inspectors, and samplers |  |
|  | Motor vehicle operators |  |
|  | Other transportation and material moving occupations |  |
|  | Construction laborers |  |
|  | Laborers, except construction |  |
|  | Freight, stock, and material movers, hand |  |
|  | Other helpers, equipment cleaners, hand packagers, laborers |  |
|  | Military occupations |  |
| Note: Due to the differences in occupational data in the NHANES database before and after 2004, we classified the data separately for the periods before and after 2004. | | |
